# Supplementary material for: The causal relationship between the human gut microbiota and pyogenic arthritis: a Mendelian randomization study
Source: Front Cell Infect Microbiol. 2024 Nov 26;14:1452480. doi: 10.3389/fcimb.2024.1452480 (PMC11629706; doi:10.3389/fcimb.2024.1452480)
Supplement: Supplementary file 1 [file DataSheet1.zip › mendelian test/ebi-a-GCST90027493.csv_forest.pdf]

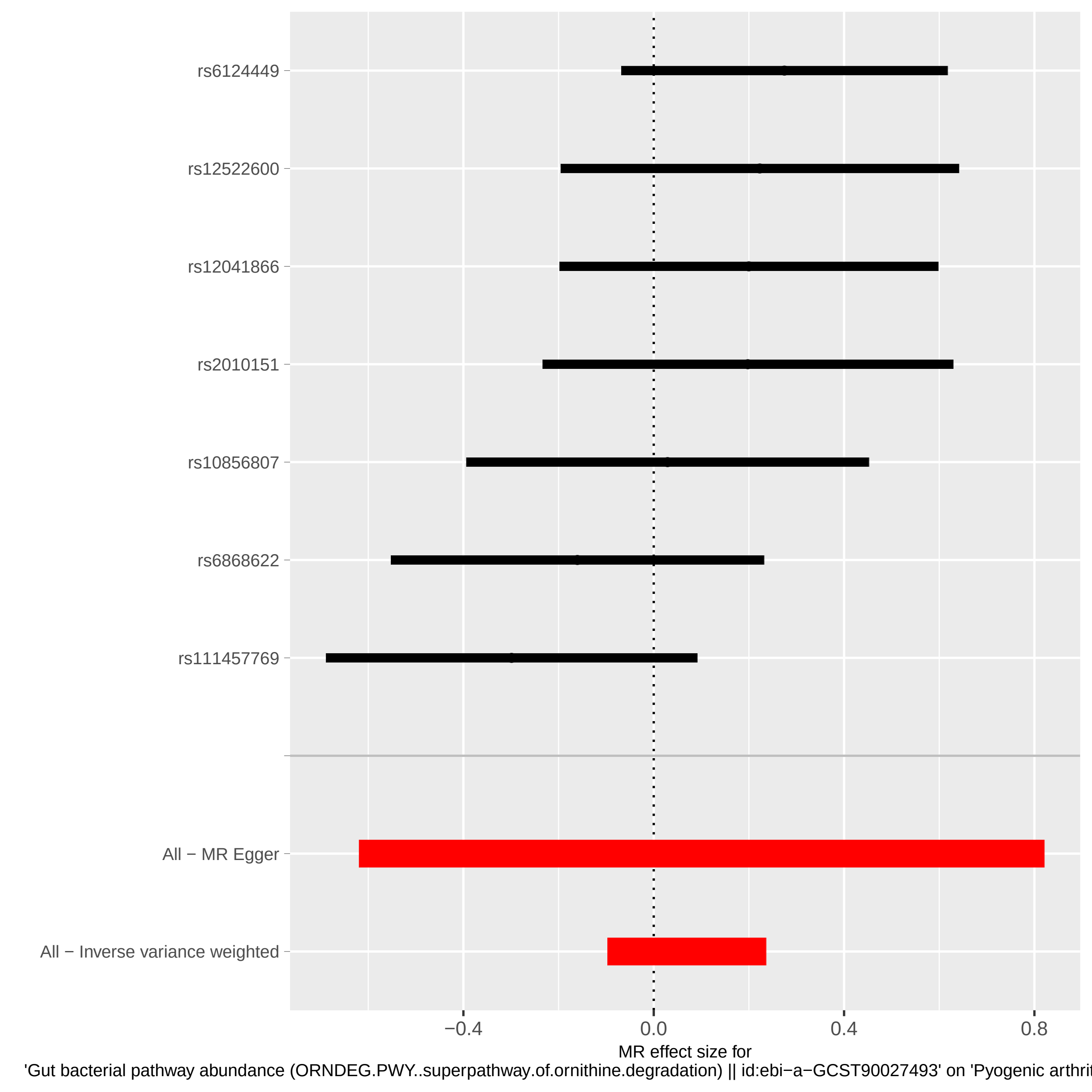

rs6124449
rs12522600
rs12041866
rs2010151
rs10856807
rs6868622
rs111457769
All − MR Egger
All − Inverse variance weighted
−0.4
0.0
0.4
0.8
MR effect size for
'Gut bacterial pathway abundance (ORNDEG.PWY..superpathway.of.ornithine.degradation) || id:ebi−a−GCST90027493' on 'Pyogenic arthrit
